# Post-COVID-19 Fatigue: Potential Contributing Factors

**DOI:** 10.3390/brainsci10121012

**Published:** 2020-12-19

**Authors:** Thorsten Rudroff, Alexandra C. Fietsam, Justin R. Deters, Andrew D. Bryant, John Kamholz

**Affiliations:** 1 Department of Health and Human Physiology, University of Iowa, Iowa City, IA 52242, USA; alexandra-fietsam@uiowa.edu (A.C.F.); justin-deters@uiowa.edu (J.R.D.); 2 Department of Neurology, University of Iowa Hospitals and Clinics, Iowa City, IA 52242, USA; john-kamholz@uiowa.edu; 3 Department of Internal Medicine, University of Iowa Hospitals and Clinics, Iowa City, IA 52242, USA; andrew-d-bryant@uiowa.edu

**Keywords:** fatigue, COVID-19, recovery

## Abstract

Much of the spotlight for coronavirus disease 2019 (COVID-19) is on the acute symptoms and recovery. However, many recovered patients face persistent physical, cognitive, and psychological symptoms well past the acute phase. Of these symptoms, fatigue is one of the most persistent and debilitating. In this “perspective article,” we define fatigue as *the decrease in physical and/or mental performance that results from changes in central, psychological, and/or peripheral factors due to the COVID-19 disease* and propose a model to explain potential factors contributing to post-COVID-19 fatigue. According to our model, fatigue is dependent on conditional and physiological factors. Conditional dependency comprises the task, environment, and physical and mental capacity of individuals, while physiological factors include central, psychological, and peripheral aspects. This model provides a framework for clinicians and researchers. However, future research is needed to validate our proposed model and elucidate all mechanisms of fatigue due to COVID-19.

## 1. Introduction

For coronavirus disease 2019 (COVID-19) patients, overcoming the acute symptoms of the disease may only be the beginning of a long and challenging path to recovery. It has been shown that after viral infections (e.g., SARS-1), patients often sustain functional limitations over a long period after discharge from the hospital. In many cases, physical, cognitive, and psychological impairments persist for multiple years [1,2]. Similarly, as COVID-19 research progresses, it has become increasingly apparent that a high proportion of patients experience persistent symptoms, such as fatigue [3,4,5].

Although the mortality rate is lower in COVID-19, it has been compared to severe acute respiratory syndrome (SARS) due to their similar symptomology [6]. Like COVID-19, persistent fatigue was apparent in SARS patients, even up to one year after their initial infection [1]. Another study showed that 40% of SARS survivors still reported fatigue after 4 years [2]. Townsend et al. [7] found that there was no association between acute symptom severity and the prevalence of persistent fatigue following COVID-19. Moreover, they showed a substantial occurrence of post-viral fatigue in individuals with previous COVID-19 disease after the acute phase of the virus.

Fatigue, which is a common and disabling symptom experienced by people with neurological disorders, remains poorly understood. Despite significant effort to explain the pathogenic mechanisms of fatigue, current knowledge is limited. Potentially, this is because the cause of fatigue cannot be narrowed down to a single source. Changes in neurotransmitter levels, inflammation, psychological disorders, stress levels, cognitive dysfunction, and substrate metabolism/availability are some potential candidates contributing to fatigue. In this perspective paper, we extend a previous model of fatigue [8] to explain factors contributing specifically to post-COVID-19 fatigue. This model was created to give researchers and clinicians an improved understanding of determinants of fatigue and provide suggestions for further research.

## 2. Definitions of Fatigue

**Figure 1:**
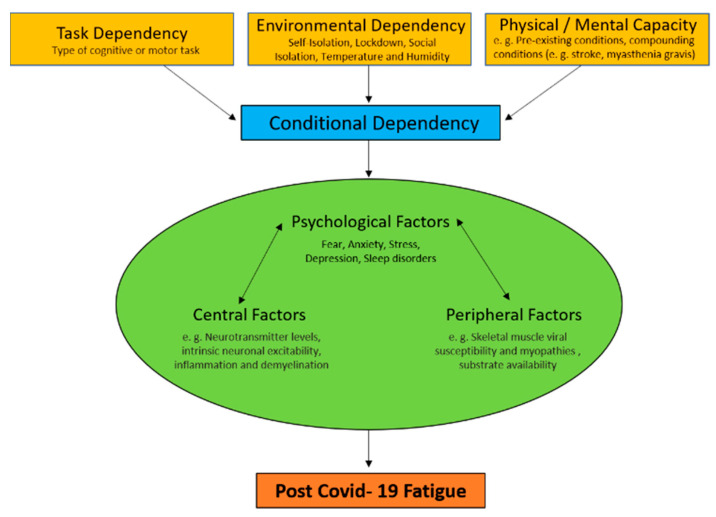
Fatigue is defined as the decrease in physical and/or mental performance that results from changes in central, psychological, and/or peripheral factors. These depend on the task being performed, the environmental conditions it is performed in, and the physical and mental capacity of the individual (conditional dependency). Importantly, fatigue is greatly affected by the factors of conditional dependency and the interactive changes in central, psychological, and/or peripheral factors (physiological factors) that cause fatigue.

Many studies investigating fatigue have failed to objectively define fatigue and existing definitions of fatigue differ significantly. Additionally, the roots of fatigue vary between conditions. Most research studies in diseases have failed to discriminate between fatigue and other confounding occurrences, such as depressed mood and sleep disorders, or consider comorbidities as contributors to fatigue. As a result, Rudroff and colleagues [8] recently proposed a unified taxonomy for fatigue in neurological disorders which, in our opinion, can be used as a template for post-COVID-19 (Figure 1). We define post-COVID-19 fatigue as *the decrease in physical and/or mental performance that results from changes in central, psychological, and/or peripheral factors due to the COVID-19 disease.* Further, we will discuss how these physiological factors may interact with the environment and task being performed to contribute further to post-COVID-19 fatigue.

## 3. Factors Contributing to Post-COVID-19 Fatigue

### 3.1. Central Factors

It is unclear if COVID-19 is neuroinvasive. However, it is suggested that other human coronavirus equivalents can use the hematogenous and neuronal dissemination to penetrate the central nervous system (CNS) [9,10,11,12]. Therefore, central factors influencing post-COVID-19 fatigue may be a result of the virus invading the CNS. *Central factors* which may contribute to COVID-19 fatigue include neurotransmitter levels (e.g., dopamine and serotonin [13,14,15,16]), intrinsic neuronal excitability, inflammation, demyelination (resulting in changes in axonal conduction velocity), and many others.

Neuroimaging studies have started to provide evidence of the central factors of fatigue in COVID-19. Delorme et al. [17] used ^18^F-fluorodeoxyglucose-positron emission tomography (FDG-PET) to measure cerebral glucose metabolism in COVID-19 patients with fatigue. They found frontal hypometabolism and cerebellar hypermetabolism, which may have an impact on fatigue. While an association between cerebral hypometabolism and fatigue has been demonstrated consistently in patients with neurological disorders [18,19,20], it cannot be ruled out that cerebral hypometabolism is caused by other symptoms, such as depression. Another FDG-PET study by Guedj et al. [21] also found significant cerebral hypometabolism in COVID-19 patients and concluded that longer follow-up studies are required to specify the association between hypometabolism and possible persistent symptoms, such as fatigue.

In addition, lockdowns across the globe have led to prolonged periods of physical inactivity becoming increasingly common. These periods of physical inactivity may cause decreased motor neuron excitability (e.g., inhibition) [22,23]. This, taken in conjunction with decreased motor unit conduction velocity as a result of COVID-19, shown by nerve conduction studies and quantitative electromyography, might additionally contribute to fatigue [24,25].

### 3.2. Psychological Factors

For many patients, COVID-19-related fatigue can simultaneously occur in an environment where stress, anxiety, depression, and fear are rampant [26]. Many measures utilized to combat the pandemic, such as quarantining, social distancing, and isolation, have proven effective at slowing the spread of the virus but may have unintended consequences that exacerbate fatigue in recovering COVID-19 patients [26,27,28]. These negative psychological consequences include post-traumatic stress symptoms, anxiety, confusion, depression, and anger. When these are taken together, it is thought they may be a significant contributor to fatigue [26,27,28]. Furthermore, in both clinical and research settings, it is also critical to distinguish COVID-19 fatigue from potentially similar symptoms, including depression, somnolence, and apathy. 

Although there may be clinical interactions between these symptoms and fatigue, they are distinct phenomena. From a research perspective, distinguishing COVID-19 fatigue from related phenomena can be accomplished by including measures of mood and sleepiness as covariates. Serotonin and dopamine are just two examples of major players in psychological fatigue. COVID-19 may get access to the brain via the forebrain’s olfactory bulb, which is rich in the neurotransmitter dopamine and is important for pleasure, motivation, and action. In addition to dopamine and serotonin, COVID-19 may alter the levels of other neurotransmitters, such as acetylcholine, which is the main cause of fatigue in myasthenia gravis [29,30]. These changes in the brain are likely responsible for the mood (e.g., stress, anxiety, and depression), fatigue, and cognitive changes that are commonly experienced by COVID-19 acute and recovered patients.

### 3.3. Peripheral Factors

Post-COVID-19 fatigue may also occur from one or several *peripheral factors*. COVID-19 may have the ability to infect a variety of tissue types, with a unique potential to target skeletal muscle. Common symptoms of COVID-19 are pain, skeletal muscle weakness, and injury occurrence [31,32,33]. Thus, it is logical to suggest that COVID-19 may directly impact skeletal muscle and, therefore, contribute to fatigue. Ferrandi et al. [34] proposed that various skeletal muscle cell types may independently and/or collectively show vulnerability to COVID-19 via angiotensin-converting enzyme 2 (ACE2). COVID-19 in the lungs activates various leukocytes to release a cascade of cytokines, including interleukin-6 (IL-6) [35]. Notably, systemic elevations of IL-6 can disrupt muscle metabolic homeostasis and exacerbate muscle loss [36]. Thus, Ferrandi et al. [34] postulate that skeletal muscle may be impacted by COVID-19 through direct infection of resident ACE2-rich cell types and/or indirectly through systemic cytokine release and subsequent homeostatic perturbation.

It should be noted that skeletal muscle myopathies are common [37] and associated with populations that are well known to be at risk for COVID-19, such as older adults [38] and patients with dystrophies [39]. Fatigue may be further compounded in older people by age-related loss of function and skeletal muscle wasting (i.e., sarcopenia) [37]. Equally significant are patients with dystrophies (e.g., Duchenne muscular dystrophy (DMD)) with system-wide skeletal muscle function loss and increased fatigability [39].

Adipose tissue may also be negatively be influenced by COVID-19. Currently, there is not strong evidence for COVID-19 disease of adipose tissue and detected virus levels in peripheral blood samples were rather low [37]. However, adipose tissue is a target for many viruses and ACE2 has been shown to be present on adipocytes [39]. Therefore, it is plausible that adipose tissue could also be targeted by COVID-19. Li et al. [32] showed an association between reduced insulin sensitivity and lower ACE2 that might be of high interest. Hyperglycemia is commonly noticed in COVID-19 disease [40] and poorly controlled glucose metabolism increases the seriousness and mortality in diabetic patients with COVID-19 [41,42,43]. With fatigue presenting as a common symptom of both diabetes and COVID-19, having both may exacerbate the occurrence of fatigue and lead to poorer long-term outcomes.

## 4. Conditional Dependency

*Conditional dependency* includes changes in central, peripheral, and psychological factors of fatigue that are dependent upon the task being performed, the environment it is performed in, and the physical and mental capacity of the individual (Figure 1). *Task dependency* has been well established as an element of fatigue in healthy and diseased individuals [44,45,46]. Human studies reveal that fatigue is not caused completely by any common set of factors alone but, rather, is dependent on the type of cognitive or motor tasks that are being performed. *Environmental dependency* reflects how factors of the environment affect fatigue in COVID-19 survivors. For example, temperature and humidity can greatly affect physical abilities of neurological patients [47]. In addition to the health-related issues and financially disturbing conditions of the COVID-19 pandemic, self-isolation, lockdown, and social isolation may have negative impacts on an individual’s *physical and mental capacity* [48]. Additionally, experiencing anxiety and distress about the pandemic while also not being physically active in quarantine may lead to increased fatigue. Pre-existing conditions should also be considered as a contributor to physical and mental capacity [49]. Many comorbidities (compounding conditions) such as cardiovascular disease, hypertension, diabetes, congestive heart failure, chronic kidney disease, chronic obstructive pulmonary disease (COPD), and cancer present with fatigue as a common symptom. This may cause these subgroups of patients to be disproportionately affected by persistent fatigue in comparison to people with no comorbidities.

### Conclusions and Future Directions

In this perspective paper, we suggested a definition of fatigue and identified factors that add to post-COVID-19 fatigue. This list is not complete; our model is hypothetical and further research is needed to explain all mechanisms of post-COVID-19 fatigue and to validate our proposed model. Research studies should concentrate on distinctly defined outcome variables that contribute to fatigue. Because of the complexity of post-COVID-19 fatigue, it is important that future studies integrate several methods directed at the different factors that influence fatigue. At this time, several methods are available to measure many of the contributing factors. Central factors can be studied via neuroimaging procedures such as FDG-PET to measure changes in glucose metabolism [50,51], psychological factors with neuropsychological tests [52], and peripheral factors can be evaluated by comparing alterations in the electromyography signal with maximal force or power output [53,54,55].
